# Television-viewing time and bodily pain in Australian adults with and without type 2 diabetes: 12-year prospective relationships

**DOI:** 10.1186/s12889-022-14566-y

**Published:** 2022-11-29

**Authors:** Francis Q. S. Dzakpasu, Neville Owen, Alison Carver, Parneet Sethi, Christian J. Brakenridge, Agus Salim, Donna M. Urquhart, Flavia Cicuttini, David W. Dunstan

**Affiliations:** 1 Mary MacKillop Institute of Health Research, Australian Catholic University, Level 5, 215 Spring Street, Melbourne, Victoria 3000 Australia; 2 Baker Heart and Diabetes Institute, Melbourne, VIC Australia; 3 Centre for Urban Transitions, Swinburne University of Technology, Melbourne, VIC Australia; 4 National Centre for Healthy Ageing, Peninsula Clinical School, Monash University, Frankston, VIC Australia; 5 Central Clinical School/Department of Epidemiology and Preventive Medicine, Faculty of Medicine, Nursing and Health Sciences, Monash University, Melbourne, VIC Australia; 6 Institute for Physical Activity and Nutrition (IPAN), School of Exercise and Nutrition Sciences, Deakin University, Geelong, VIC Australia

**Keywords:** Bodily pain trajectory, Chronic pain, Growth curve model, Prediabetes, Sedentary behaviour, TV time, Type 2 diabetes

## Abstract

**Background:**

Bodily pain is a common presentation in several chronic diseases, yet the influence of sedentary behaviour, common in ageing adults, is unclear. Television-viewing (TV) time is a ubiquitous leisure-time sedentary behaviour, with a potential contribution to the development of bodily pain. We examined bodily pain trajectories and the longitudinal relationships of TV time with the bodily pain severity; and further, the potential moderation of the relationships by type 2 diabetes (T2D) status.

**Method:**

Data were from 4099 participants (aged 35 to 65 years at baseline) in the Australian Diabetes, Obesity and Lifestyle Study (AusDiab), who took part in the follow-ups at 5 years, 12 years, or both. Bodily pain (from SF36 questionnaire: a 0 to 100 scale, where lower scores indicate more-severe pain), TV time, and T2D status [normal glucose metabolism (NGM), prediabetes, and T2D] were assessed at all three time points. Multilevel growth curve modelling used age (centred at 50 years) as the time metric, adjusting for potential confounders, including physical activity and waist circumference.

**Results:**

Mean TV time increased, and bodily pain worsened (i.e., mean bodily pain score decreased) across the three time points. Those with T2D had higher TV time and more-severe bodily pain than those without T2D at all time points. In a fully adjusted model, the mean bodily pain score for those aged 50 years at baseline was 76.9(SE: 2.2) and worsened (i.e., bodily pain score decreased) significantly by 0.3(SE: 0.03) units every additional year (*p* <0.001). Those with initially more-severe pain had a higher rate of increase in pain severity. At any given time point, a one-hour increase in daily TV time was significantly associated with an increase in pain severity [bodily pain score decreased by 0.69 (SE: 0.17) units each additional hour; *p* <0.001], accounting for the growth factor (age) and confounders’ effects. The association was more-pronounced in those with T2D than in those without (prediabetes or NGM), with the effect of T2D on bodily pain severity becoming more apparent as TV time increases, significantly so when TV time increased above 2.5 hours per day.

**Conclusion:**

Bodily pain severity increased with age in middle-aged and older Australian adults over a 12-year period, and increments in TV time predicted increased bodily pain severity at any given period, which was more pronounced in those with T2D. While increasing physical activity is a mainstay of the prevention and management of chronic health problems, these new findings highlight the potential of reducing sedentary behaviours in this context.

**Supplementary Information:**

The online version contains supplementary material available at 10.1186/s12889-022-14566-y.

## Background

Bodily pain increases with age and can be of somatic, visceral, or neurogenic origin [1, 2]. Among Australian adults aged 45-years and over, it has been estimated that 20% experience persistent chronic pain [3]. The challenges to clinical management and public health implications of chronic pain are substantial and often associated with multimorbidity, including diabetes and cardiovascular disease (CVD). Furthermore, those with diabetes can be more likely to be hospitalized for musculoskeletal pain-related conditions [4]. Chronic pain impacts adversely on daily physical activity and quality of life; can be associated with physical and mental health problems; and, substantially contributes to healthcare costs and the economic burden of lost productivity [5].

The prevalence and burden of chronic pain both increase with advancing age and as physical activity participation declines [6]. Chronic pain can be associated with older adults being physically inactive and large amounts of time sitting. While changes in physical activity with advancing age have been studied extensively [7, 8], recent research attention has been directed at increases in sedentary behaviour (which is distinct from physical inactivity, and defined as time spent in a sitting or reclining posture with energy expenditure less than 1.5METs) [9]. Higher volumes of sedentary time can be associated with increased risk of all-cause mortality, incident CVD, type 2 diabetes (T2D), and some cancers [10–13]. Specifically, one of the most common leisure-time sedentary behaviours – television-viewing (TV) time – has been consistently shown to be associated with multiple adverse chronic health outcomes [12–16], providing a simple, self-report indicator of a common domain-specific sedentary behaviour in community-based adults in the home settings [17].

There is evidence of detrimental associations of higher volumes of TV time with the risk of developing chronic diseases such as CVD, T2D, musculoskeletal disorders, and some cancers which is important in this context [10, 13, 15], as well as an adverse impact on physical activity levels in ageing adults [18]. However, there is limited evidence on the influence of prospective changes in TV time on bodily pain trajectories with ageing.

In epidemiological studies of sedentary behaviour and pain, the 36-Item Short-Form Health Survey (SF-36) questionnaire [19] has been commonly used, with mixed evidence on associations with bodily pain scale scores [20, 21]. To date, only a few prospective studies, typically in small subgroups of adults, have investigated longitudinal associations between TV time and pain, with inconsistent findings [21, 22]. Large cohort studies are yet to examine prospective relationships of changes in TV time with bodily pain trajectories. Further, the effects of sedentary behaviour can be more pronounced in those with metabolic disorders, particularly in T2D which is a major risk factor of CVD [23–25]. For example, a review of experimental and intervention-trial evidence has shown that reducing sedentary behaviour can beneficially impact cardiometabolic and inflammatory biomarkers associated with T2D [25]. Also, T2D has been shown to be associated with heightened chronic pain conditions, especially neuropathic pain [26–28]. Since studies have also shown that sedentary time is more pronounced in those with T2D compared to those without [29], there is a need to better understand the convergence of high sedentary time with T2D on trajectories of bodily pain. Specifically, it is unknown whether the potential influence of TV time on prospective changes in bodily pain differs according to the presence or absence of T2D.

We examined the longitudinal relationships of concurrent changes in TV time with bodily pain at three observation points over 12 years in Australian adults who were middle-aged and older at baseline; and, whether such potential relationships may be moderated by T2D status. We hypothesized that bodily pain severity would increase with age. Also, increasing TV time would be associated with increased severity of bodily pain at any given time point, and the strength of the association would differ between those with T2D and those without T2D.

## Methods

### Study sample and participant selection

The Australian Diabetes, Obesity, and Lifestyle Study (AusDiab), a general population-based study of community-dwelling Australian adults aged ≥25 years to describe diabetes prevalence and cardiometabolic risk markers, was initiated in 1999/2000 (baseline – Wave 1), with two subsequent follow-ups in 2004/05 (Wave 2) and 2011/12 (Wave 3). Description of the study design and participants has been published elsewhere [30]. Initially, baseline data (*n* = 11,247) were collected from adults residing in 42 Australian Bureau of Statistics Census Collector District (CCD) across all States and the Northern Territory. Those with physical or intellectual disabilities were not included [30]. The first follow-up at five years (*n* = 8798), was undertaken in 2004/05; and the second follow-up at 12 years (*n* = 6,186), in 2011/12 as detailed elsewhere [31, 32]. At each respective time point, interviewer and self-administered questionnaire data, as well as biomedical data, including physical examination, urine and blood samples were collected at a local testing site [30–32]. The study was approved by the International Diabetes Institute (now Baker Heart and Diabetes Institute) Ethics Committee and the Alfred Ethics Committee, project approval no. 39/11.

**Fig. 1:**
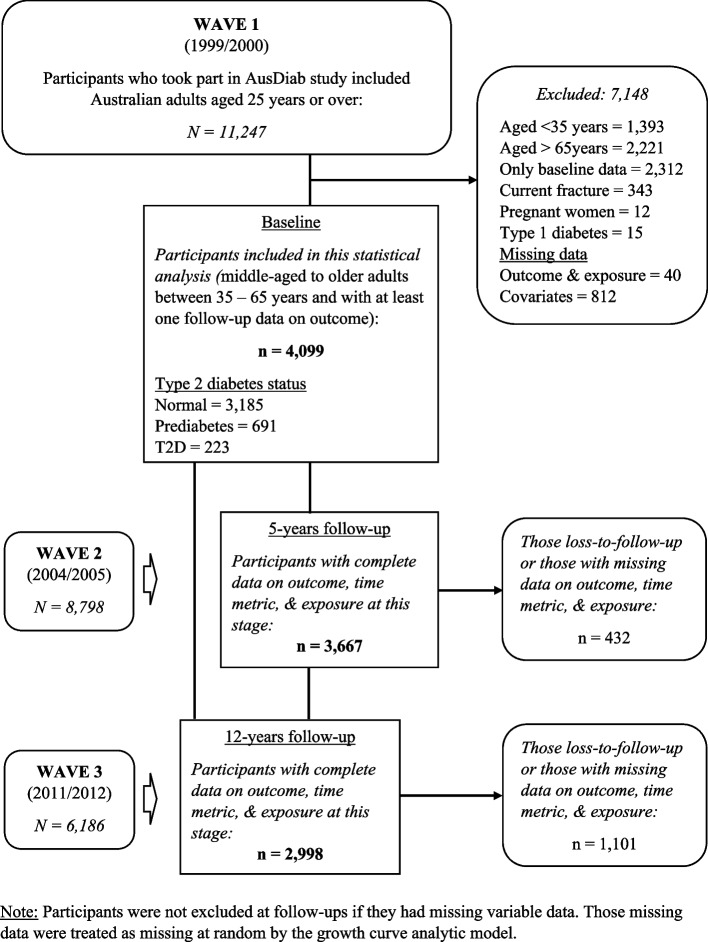
A flowchart diagram of participants at baseline and consecutive follow-ups

For this analysis, we considered the middle-aged and older participants aged 35 to 65 years with and without T2D at baseline. This was based on recent findings reported by the Australian Institute of Health and Welfare suggesting that one in five Australian adults aged 45 years and over live with chronic pain with physical inactivity, smoking, overweight, and obesity as the likely associated behavioural risk factors [3]. Those with type 1 diabetes, a history of current bone fracture, and women who were pregnant were excluded from the analyses. Initially, the 4099 participants who were considered for inclusion in these analyses had complete data for the outcome, exposure, and all relevant covariates variables at baseline and at least one instance of follow-up data for SF-36 bodily pain, TV time, leisure-time physical activity, and T2D status. Among these participants, a total of 223 participants were categorised as having T2D based on self-reported T2D status (101) and a newly clinically determined T2D status (122) based on a fasting blood glucose test or 2-hour oral glucose tolerance test (OGTT); 691 as prediabetes [impaired fasting glucose (IFG) or impaired glucose tolerance (IGT)]; and 3,185 as normal glucose metabolism (NGM). The total number of participants included in the analysis based on our selection criteria and those excluded at baseline, as well as the number remaining and those loss-to-follow-up at the 5-year and 12-year time points are illustrated in a flowchart in Fig. 1.

### Variables

#### Outcome: bodily pain

The bodily pain scores were derived at all data time-points from the validated 36-item Short Form (SF-36) self-report survey instrument for assessing health-related quality of life (HRQoL) [19, 33]. Two of the SF-36 items (items 7 and 8) measure bodily pain dimensions - the intensity and the extent of interference with daily activity (based on a standard SF-36 questionnaire 4-week recall of chronic/persistent pain) [19, 34]. Item 7 asked: “How much bodily pain have you had during the past 4 weeks?” with the response options: “1 = None; 2 = Very mild; 3 = Mild; 4 = Moderate; 5 = Severe; 6 = Very severe”. Item 8 asked: “During the past 4 weeks, how much did pain interfere with your normal work (including both work outside the home and housework)?”, and the options were: “1 = Not at all; 2 = A little bit; 3 = Moderately; 4 = Quite a bit; 5 = Extremely”. A validated scoring algorithm was used to transform the two items' responses into a single bodily pain score on a 0 to 100 scale [19], whereby the lowest possible score of “0” indicates severe bodily pain and the highest possible score of “100” indicates no bodily pain [33]. The accuracy of the SF-36 instrument to estimate HRQoL is high, with acceptable psychometric properties across all the measured dimensions in different demographic, health-related behaviour risk factors, and socioeconomic population groups in Australia [35]. A validation study in Australia indicated the 2-item bodily pain dimension has a high homogeneity (item-correction = 0.95) and internal consistency (Cronbach alpha = 0.90) [35].

#### Exposure: television-viewing time

The main explanatory variable (time spent watching television – TV time) was assessed at each time point. Participants self-reported total time spent on each weekday and weekend day watching television or video/DVD for the past week, excluding times when the television was switched on, but other leisure-time activities were being concurrently undertaken [12]. The total daily TV time was estimated by averaging the duration of TV time across seven days (the five weekdays and two weekend days) in hours. Psychometric studies indicate that this measure of TV time has acceptable properties in adults, with moderate-to-high validity and reliability, with a test-retest reliability intraclass correlation coefficient (ICC) of 0.66 (95% CI = 0.50 – 0.83) [17] and a Spearman correlation of 0.3 for a 3-day behavioural log criterion validity [36].

#### Moderator: type 2 diabetes status

T2D status was ascertained from self-reported data at baseline for known diabetes and by clinical diagnosis based on the standard recommended World Health Organisation (WHO) fasting blood/plasma glucose (FBG) test and 2-hour OGTT at each data time-point [37]. The T2D status variable was grouped into four categories (NGM, prediabetes, new T2D, and known T2D). The newly diagnosed T2D at each wave became known T2D at the subsequent wave. T2D was defined as FBG greater than 7.0 mmol/L or 2-hour OGTT greater than 11.1mmol/L. Prediabetes was defined according to the American Diabetes Association (ADA) criteria as IFG if FBG was in the range of 5.6 – 6.9 mmol/L or IGT if 2-hour OGTT fell in the range of 7.8 –11.0 mmol/L; NGM was defined as FBG less than 5.6 mmol/L and 2-hour OGTT less than 7.8 mmol/L [37]. If there were missing data on any one of the assessment methods (either FBG or 2-hour OGTT), the classification of NGM was based on the non-missing data.

#### Covariates

Potential confounding time-invariant variables (attributes that varied between participants but remained unchanged at the data time-points) included sex and education level were captured only at baseline. Additionally, time-variant confounders which differed between participants, and also changed within participants at the data time points were considered. These included participants’ age, and waist circumference measured in centimetres (cm). Further, leisure-time physical activity time was assessed using the Active Australia Survey (AAS) instrument [38] to capture participants’ time spent in moderate-to-vigorous intensity physical activity (MVPA) at the three-time points. The AAS predominantly measures leisure-time physical activity according to the domain in which it took place and includes time spent walking for transport and leisure; moderate-intensity physical activity; and vigorous-intensity physical activity in the past week. The total physical activity time was estimated as the sum of time spent walking continuously for 10 or more minutes for transport or recreation plus time spent in moderate-intensity physical activity plus twice the time spent in vigorous-intensity physical activity. The calculation also accounts for higher energy expenditure associated with vigorous-intensity physical activity per unit time [38, 39]. The AAS instrument has an acceptable psychometric test-retest reliability ICC = 0.64; CI = 0.57 – 0.70) [40], and also acceptable validity against accelerometer-estimated physical activity (Spearman correlation = 0.61; CI = 0.43 – 0.75) [41].

Other time-varying confounders were participants' self-reported household income, and some relevant lifestyle behaviours including total energy intake, and smoking (three categories - never smoked, ex-smoker, and current smoker). Also, confounders related to the medical status included self-reported SF-36 mental component score, clinically assessed chronic kidney disease (CKD) based on estimated glomerular filtration rate (yes/no), history of cancer (yes/no: note that data was available at baseline and was treated as a time-invariant variable), and history of CVD which included angina, coronary heart disease, heart attack, or stroke (yes/ no).

### Statistical analysis

All analyses were performed using STATA statistical software (version 14.2; StataCorp LLC) and the findings were deemed statistically significant at p ≤ 0.05. Participants' characteristics were described across the three data time points in summary statistics. Continuous variables were presented as mean values with standard deviations; categorical variables were in proportion. We used Box plots to illustrate the differences in the bodily pain score and TV time variables according to T2D status at the various data time points. Also, mixed-effects regression was used to examine the differences in the mean bodily pain score and mean TV time across the data time points in the overall sample and according to T2D status – NGM, prediabetes and T2D (newly diagnosed and known T2D combined). Confounders were selected based on prior literature; the outcome variable (bodily pain score) was regressed with all potential covariates, and multicollinearity was tested by Variance Inflation Factor (VIF > 10).

The bodily pain trajectory with age was examined by a multilevel linear growth curve model, an ideal approach for longitudinally structured data [42, 43], considering the continuous nature of the repeated measured bodily pain score. The bodily pain trajectory was modelled using participants' age at the three data time points as the time metric. Progressively adjusted models were fitted, starting with an unconditional growth (bodily pain) trajectory (Model 1) by regressing bodily pain score as a function of age (centred at age 50 years, about the mean age at baseline) using a random slope model, a more flexible growth curve modelling which estimates both intercept variance and slope variance, as well as intercept-slope covariance. The model selection and equations for the unconditional growth curve are provided in the Supplementary File.

First, the relationship between TV time and the bodily pain trajectory was examined by conditioning the bodily pain trajectory on TV time – a continuous variable in hours/day – as an exposure variable was fitted as a time-varying variable (Model 2). To understand whether the effect of TV time on bodily pain trajectory changed with age, a TV time/age interaction term was added to the fitted model, but the interaction term was statistically non-significant. A linear-additive model was therefore fitted, excluding the interaction term. The fitted model was fully adjusted for other covariates: sex, waist circumference, education level, income, energy intake, leisure-time physical activity, smoking status, T2D status, CKD, SF-36 mental component score, history of CVD, and history of cancer (Model 3).

Second, to examine the potential moderation of the relationship between TV time and bodily pain trajectory by T2D status, a multiplicative interaction between TV time and T2D status was modelled. Three categories of T2D status [NGM, pre-diabetes, and T2D (new T2D and known T2D combined)] were used in the regression models for ease of interpretation. A full interaction of TV time with T2D status was added to the fitted unconditional model (Model 1); predictive margins and marginal effects (the impact T2D status has on the changes in bodily pain severity $\left[\frac{\partial bodily\ pain\ score}{\partial T2D\ status}\right]$ when TV time is held constant at different points or thresholds) with standard errors estimated and outputs illustrated in a line graph (Model 4) [44]. Finally, the fitted model was fully adjusted for sex, waist circumference, education level, income, energy intake, leisure-time physical activity, smoking status, CKD, SF-36 mental component score, history of CVD, and history of cancer; predictive margins as well as marginal effects and standard errors were estimated, and results illustrated in a line graph (Model 5).

#### Sensitivity analysis

Two sensitivity analyses were performed to check the robustness of our analysis. First, we performed a sensitivity analysis by excluding data for those who reported a history of cancer. Data on participants’ history of cancer was only available at a one-time point (baseline) with the assumption made that it was a time-invariant covariate in the analysis. Secondly, many of those with a history of cancer may be more likely to self-report experiencing more pain. Therefore, the sensitivity analytic sample comprised the remaining 3827 participants with complete data at baseline. A second sensitivity analysis was performed using data from only those participants who provided data at baseline and both of the respective follow-ups. A total of 2727 participants’ data were modelled in this sensitivity analysis, adjusting for all covariates described for the main analysis, including the history of cancer variable.

## Results

Participant characteristics are presented in both Tables 1 and 2. The mean age at baseline was 49.4 ± 8.0 years, and the average bodily pain score decreased (i.e., bodily pain worsened) from baseline through 5-year follow-up to the 12-year follow-up (p <0.001). Mean TV time increased significantly across the three time points (p <0.001). The proportion of participants with T2D (newly diagnosed and known T2D) increased from 5.5% at baseline to 9.3% and 13.2% at 5-year and 12-year follow-ups, respectively.

**Table 1:** Participants’ characteristics across the data time-points

Variables	Baseline		5-year Follow-up		12-year Follow-up	
	N	Mean (SD) or %	N^b^	Mean (SD) or %	N^b^	Mean (SD) or %
**Time metric**						
*Age, years*	4099	49.42 (7.99)	3693	54.58 (8.02)	3085	60.96 (7.86)
**Outcome**						
*SF36 bodily pain score*	4099	75.51 (21.75)	3694	74.49 (22.36)	3124	72.91 (22.08)
**Exposure variable**						
*TV time, hrs/day*	4099	1.69 (1.24)	3674	1.86 (1.29)	3010	1.92 (1.32)
**Moderator: T2D Status**						
*NGM*	3185	77.7%	2952	78.6%	2634	72.4%
*Prediabetes*	691	16.9%	454	12.1%	522	14.4%
*New T2D*	122	3.0%	80	2.1%	62	1.7%
*Known T2D*	101	2.5%	272	7.2%	418	11.5%
**Covariates**						
Sex^a^						
*Female*	2227	54.3%	2007	54.4%	1709	54.7%
*Male*	1872	45.7%	1685	45.6%	1415	45.3%
Waist circumference, cm	4099	90.22 (13.77)	3689	92.47 (13.97)	3082	95.13 (14.24)
MVPA, min/week	4099	282.27 (334.06)	3673	299.93 (325.50)	3477	337.96 (357.77)
Education level^a^						
*At least college*	1439	35.1%	1294	35.0%	1178	37.7%
*Below college*	2660	64.9%	2400	65.0%	1946	62.3%
House income						
*High*	2934	71.6%	2697	74.0%	2255	74.9%
*Low*	1127	27.5%	909	24.9%	510	16.9%
*Not provided*	38	0.9%	39	1.1%	246	8.2%
Energy intake, kcal	4099	8119.38 (3281.22)	3641	7639.79 (3070.71)	2992	7139.74 (2827.59)
Smoking status						
*Current smoker*	486	11.9%	335	9.4%	204	6.0%
*Ex-smoker*	1226	29.9%	1135	32.0%	1232	36.0%
*Non-smoker*	2387	58.2%	2078	58.6%	1984	58.0%
SF36 MCS	4099	49.15 (9.41)	3693	49.66 (9.62)	3102	57.53 (12.02)
Chronic kidney disease						
*No*	3927	95.8%	3531	95.2%	3086	97.8%
*Yes*	172	4.2%	179	4.8%	71	2.3%
History of CVD						
*No*	3953	96.4%	3790	95.3%	3279	92.0%
*Yes*	146	3.6%	188	4.7%	284	8.0%
History of cancer^a^						
*No*	3827	93.4%	3457	93.6%	2914	93.3%
*Yes*	272	6.6%	237	6.4%	210	6.7%

^a^ Time invariant variable, *N* Total number of participants, *SD* Standard deviation, *TV* Television-viewing, *NGM* Normal glucose metabolism, *T2D* Type 2 diabetes, *MVPA* Moderate-to-vigorous intensity physical activity (leisure-time physical activity), *CS* Mental Component Score, *CVD* Cardiovascular diseases

^b^Participants with non-missing data for any of the variables at follow-ups were included in the data presented in this descriptive table

**Table 2:** Mean bodily pain score and TV time across the data time-points

Parameters	Baseline	5-year Follow-up	12-year Follow-up	*P*-value
**Bodily pain score,** *mean (SD)*				
Overall sample	75.51 (21.75)	74.49 (22.36)	72.91 (22.08)	<0.001
NGM	76.12 (21.15)	75.85 (21.61)	74.29 (20.03)	<0.001
Prediabetes	74.24 (23.47)	71.35 (23.19)	72.27 (23.46)	0.078
T2D	70.87 (24.05)	67.17 (25.46)	65.54 (25.17)	<0.001
**TV time (hrs/day), ***mean (SD)*				
Overall sample	1.69 (1.24)	1.86 (1.29)	1.92 (1.32)	<0.001
NGM	1.62 (1.20)	1.80 (1.27)	1.83 (1.28)	<0.001
Prediabetes	1.84 (1.36)	2.02 (1.36)	2.13 (1.39)	<0.001
T2D	2.18 (1.30)	2.21 (1.38)	2.26 (1.39)	0.112

*NGM* Normal glucose metabolism, *T2D* Type 2 diabetes (included new T2D and known T2D), *SD* Standard deviation, *TV* Television-viewing

**Fig. 2:**
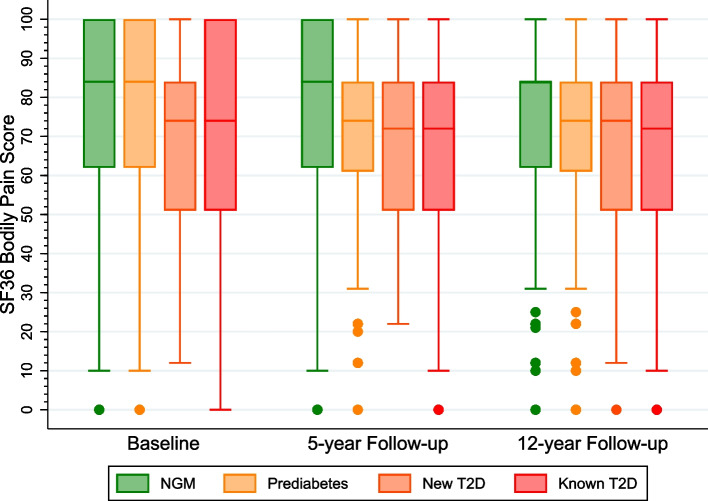
Shows box plots comparing the mean bodily pain score according to type 2 diabetes (T2D) status [normal glucose metabolism (NGM), prediabetes, new T2D, and known T2D] at the three time points. **Note:** Higher score means less pain and a lower score indicates severe pain. The dots indicate outliers.

**Fig. 3:**
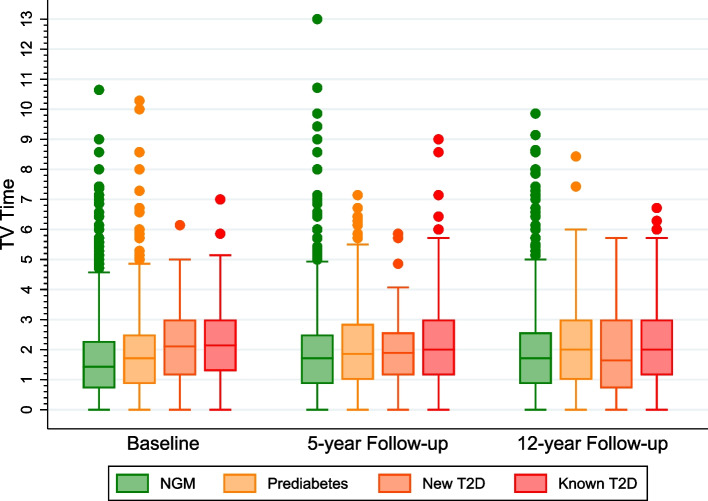
Shows box plots comparing the mean television-viewing (TV) time according to T2D status (NGM, prediabetes, new T2D, and known T2D) at the data time points. Note: The dots indicate outliers.

As illustrated in the box plots in Figs. 2 and 3, those with T2D, particularly those with known T2D had relatively more severe pain. The known T2D group had relatively higher mean TV time at each data time point than the other groups, but these were not statistically-significant differences.

As shown in Table 2, the increase in the severity of bodily pain across the three-time points was statistically significant among those with NGM and T2D (p <0.001), but marginally non-significant in the prediabetes group (p <0.078). The differences in the mean TV time at the three data time points were statistically-significant in only those participants with NGM and pre-diabetes (p <0.001).

### Unconditional growth (bodily pain) trajectory

The unconditional growth curve model output is shown in Table 3. The average estimated mean bodily pain score for participants aged 50 years at baseline was 75.6 (SE: 0.5), which significantly decreased (i.e., pain severity worsened) at a rate of 0.28 (SE: 0.02) unit points every additional year. There were, however, significant variations in the bodily pain scores of participants aged 50 years at baseline after accounting for the clustering of participants. The significant estimate of a positive intercept-slope covariance and negative slope for age 50 (the time metric) implies that those with higher baseline bodily pain scores (less pain) tend to have a below-average rate of decline in their bodily pain score with increasing age. Conversely, those with severe pain (low bodily pain score) at baseline tended to experience increasing pain severity (higher rate of decrease in bodily pain score) with increasing age.

**Table 3:** Unconditional and conditional linear growth curve models for bodily pain

	Unconditional model	Conditional models			
	Model 1: (Function of age)	Model 2: Model 1 + TV time	Model 3: Fully adjusted linear-additive model	Model 4: Model 2 + TV time # T2D status	Model 5: Fully adjusted with TV time#T2D status
	*Coefficient (S.E)*	*Coefficient (S.E)*	*Coefficient (S.E)*	*Coefficient (S.E)*	*Coefficient (S.E)*
**Fixed effect**					
Intercept	75.55 (0.45)***	77.53 (0.52)***	76.92 (2.20)***	77.83 (0.54)***	76.71 (2.20)***
Slopes					
Age (Centred at 50 years)	- 0.28 (0.02)***	- 0.24 (0.02)***	- 0.30 (0.03)***	- 0.21 (0.03)***	- 0.30 (0.03)***
TV time		- 1.15 (0.17)***	- 0.69 (0.17)***	- 1.03 (0.19)***	- 0.56 (0.19)**
T2D status					
NGM (Reference)				0	0
Prediabetes				- 0.97 (0.96)	0.91 (0.96)
T2D				- 4.07 (1.50)**	0.53 (1.48)
TV time#T2D status					
NGM (Reference)				0	0
Pre-diabetes				- 0.10 (0.40)	- 0.22 (0.40)
T2D				- 0.63 (0.56)	- 0.97 (0.55)^$^
**Random effect**					
Intercept variance					
Cluster^a^	4.85 (1.92)**	4.09 (1.75)**	1.05 (0.87)	3.49 (1.59)**	1.03 (0.87)
Participants	192.89 (7.48)***	191.05 (7.46)***	145.05 (6.05)***	188.80 (7.42)***	145.05 (6.05)***
Slope variance	0.014 (0.006)**	0.015 (0.006)**	0.007 (0.001)**	0.015 (0.006)**	0.008 (0.001)**
Intercept-Slope covariance	1.65 (0.34)**	1.69 (0.34)**	1.04 (0.06)***	1.66 (0.34)**	1.04 (0.06)***
Within-individual variance	266.03 (4.58)***	265.65 (4.61)***	263.06 (4.75)***	265.56 (4.61)***	262.94 (4.74)***
**Goodness-of-fit**					
AIC	95971.21	95117.23	90691.72	95024.25	90692.51
BIC	96022.25	95175.50	90858.33	95111.64	90873.61
Log-likelihood	- 47978.61	- 47550.62	- 45322.86	- 47500.12	- 45321.26
No of parameters	7	8	23	12	25

Statistically significant: *** *p* < 0.001, ** *p* < 0.01, * *p* < 0.05, ^$^ *p* = 0.076

*TV time#T2D Status* Interaction between TV time and T2D status, *TV* Television-viewing, *S.E* Standard error, *NGM* Normal glucose metabolism, *T2D* Type 2 diabetes (included newly diagnosed and known T2D)

The fully adjusted linear additive model 3 included model 2 + sex, education level, household income, smoking status, leisure-time physical activity, waist circumference, energy intake, T2D status, SF36 mental component score, presence of chronic kidney disease, history of cardiovascular disease (CVD), and cancer.

The fully adjusted model 5 with TV time#T2D status included model 4 + sex, education level, household income, smoking status, leisure-time physical activity, waist circumference, energy intake, SF36 mental component score, presence of chronic kidney disease, history of cardiovascular disease (CVD), and cancer.

^a^ This represents the intercept variance that is attributable to the level 3 clustering of individuals (individuals nested in clusters); thus, describes the variance component of cluster-to-cluster variability.

### Relationship of TV time with the bodily pain trajectory at a given time point

The conditional growth trajectory models are also presented in Table 3. A one-unit (one-hour) increase in TV time per day significantly predicted a 1.15 (SE: 0.17) point decrease in bodily pain score (thus, increase in bodily pain severity) at any given time point (e.g., at age 50 years), after accounting for the linear change in age — the growth factor (Model 2). Compared to the unconditional model (Model 1), conditioning on (i.e., adjusting for) TV time in Model 2 increased the mean baseline bodily pain score [77.5 (SE: 0.5)] at age 50 years; also, the slope variance for age 50 increased by 7.1%.

The fully-adjusted model showed that the estimated mean bodily pain score at baseline for those aged 50 years was 76.9 (SE: 2.2) (Model 3). With all other covariates held constant, the rate of increasing bodily pain severity with age (the yearly increase) was significantly estimated as 0.30 (SE: 0.03), a slight increase compared to 0.28 (SE: 0.02) of the unconditional growth model (Model 1). The slope variance for age, however, decreased by 50.0% compared to the unconditional growth model. The linear-additive marginal effect of TV time on bodily pain severity at any given time point reduced from 1.15 (SE: 0.17) in Model 2 to 0.69 (SE: 0.17) in Model 3 but remained statistically significant (p <0.001). The intercept-slope covariance was positive and remained statistically significant, meaning that those with initial more-severe pain at baseline have a significantly higher rate of increasing bodily pain severity with advancing age.

### Moderation of the relationship between TV time and bodily pain severity by T2D status

**Fig. 4:**
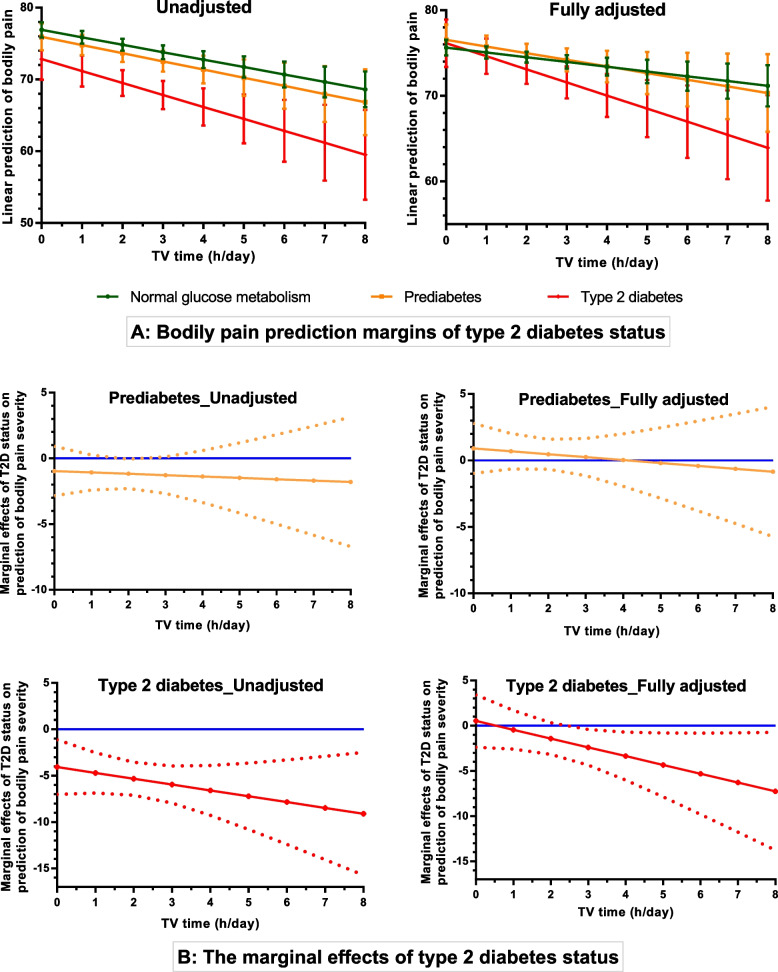
This shows the relationships of TV time with bodily pain severity and potential moderation of T2D status. (**A**) The bodily pain prediction margins of T2D status with 95% confidence intervals for the unadjusted and fully adjusted models. (**B**) The marginal effects of prediabetes and T2D (in reference to NGM) on bodily pain severity at different TV time thresholds for the unadjusted and fully adjusted models. The solid lines indicate the marginal effects of changes in bodily pain severity with changing TV time. The dotted lines are the confidence intervals around the lines, which determine the threshold of TV time that has a statistically significant effect on bodily pain severity in those with prediabetes (ORANGE) and T2D (RED). They are statistically significant whenever the lower and upper limits of the confidence intervals are both below or above the zero (0 - BLUE) lines. Note: NGM was set as the reference point in the regression model.

Models 4 and 5 in Table 3, as well as Fig. 4, show the relationships of the multiplicative interaction between TV time and T2D status with bodily pain trajectory. For those with NGM, the marginal effect of prediabetes and T2D were negative in the unadjusted Model 4 but positive in the fully adjusted Model 5. These indicate that when TV time was zero (0) in Model 4 bodily pain severity was significantly higher in the T2D but non-significant for prediabetes compared to NGM (negative coefficients – increased bodily pain severity); however, after accounting for the confounding effects of other covariates in Model 5, changes in bodily pain severity were non-significant (positive coefficients – less bodily pain severity) in both prediabetes and T2D when TV time was equal to zero (0). The interaction terms in Model 4 were non-significantly negative for both prediabetes and T2D, and in Model 5, the interaction terms remained negative but marginally non-significant for T2D [- 0.97 (SE: 0.55); p = 0.076] and non-significant for prediabetes. Thus, the severity of bodily pain with increasing TV time in the NGM, prediabetes, and T2D groups was different and more pronounced in the T2D group as illustrated in Fig. 4A. Furthermore, compared to the NGM, the effect of T2D and prediabetes on bodily pain severity (decreasing bodily pain score) increased as TV time increases. This was observed to be statistically significant for T2D but not prediabetes when the volume of TV time increased more than 2.5 hours per day (Fig. 4B – the fully adjusted model).

### Sensitivity analysis

For the first sensitivity analysis, after excluding participants with a history of cancer (due to the increased potential to self-report pain) from the analysis, similar results were observed with only slight changes in the effect sizes (results provided in Supplementary file, Table S1). However, the marginal non-significant TV time and T2D interaction term in the fully-adjusted model 5 was attenuated, but the trend of the bodily pain severity with increasing TV time for the different T2D status groups, as well as the effect of T2D on bodily pain severity with increasing TV time remained (results provided in Supplementary file, Figure S1).

Similar results were observed in the second sensitivity analysis performed on those participants with data at baseline and both of the follow-ups. There were only slight changes in the effect sizes, but the trends remained (Supplementary file, Table S2). The main difference observed was the statistically significant interaction of TV time with T2D in model 5 for the sensitivity analysis (Supplementary file, Table S2, p < 0.05) but marginally non-significant in the main analysis (Table 3, p = 0.076). Also, in this second sensitivity analysis, the effect of T2D on bodily pain severity was significantly pronounced when the threshold of TV time increased above 3 hours per day (Supplementary file, Figure S2).

## Discussion

This study examined the relationships of concurrent changes in TV time with bodily pain in a large cohort study of Australian middle-aged to older adults with and without T2D over a 12-year period. We found that bodily pain severity increased with age, and that increasing TV time at a given time point was significantly associated with increased severity of the bodily pain which persisted after adjustment for relevant confounders, including leisure-time physical activity and waist circumference. The relationships of increasing TV time with bodily pain severity at a given time point on the bodily pain trajectories were more pronounced in those with T2D than in those without T2D (prediabetes or NGM). The effect of T2D on bodily pain severity was more apparent when the threshold of TV time increased above 2.5 hours per day.

The findings corroborate some previous evidence, as well as providing novel insights into the prospective associations of sedentary behaviour with pain conditions [20, 22]. A previous epidemiological study of community-dwelling older adults, for example, identified a prospective association of self-reported higher sitting time with worse bodily pain [20]. Similarly, a prospective study of Brazilian schoolteachers found an association between increased TV time and musculoskeletal pain [22]. Although our findings are consistent with those of previous studies, we report the first evidence of an increase in severity of bodily pain with advancing age in middle-aged and older adults with increasing hours per day spent watching television at any given period. Also, we identified the moderation of this relationship by T2D status, which has not previously been reported. Our findings suggest that the magnitude of the detrimental relationships of higher volumes of TV time with bodily pain severity at any given time point is different in those with and without T2D. These findings may have potentially-different clinical and public health implications in these populations. For example, those with T2D may have a raised possible risk of a “vicious cycle”, especially in those with comorbid chronic pain; this could result in higher volumes of sedentary behaviours (including more time sitting watching television), which could worsen the severity of both T2D and pain.

In contrast, a previous study has also reported no evidence of a prospective association between sedentary behaviour, specifically, TV time and SF36 questionnaire-assessed bodily pain, albeit in a disease-specific population of cancer survivors [21]. Compared to our study, aside from the differences in the studied population, this previous prospective study [21] used a “changed analysis” approach to examine data from two time-points over 10 years, whereas our analysis was based on the multilevel growth curve approach to analyse three time-points data over 12-years. The differences in the analytical approach and study populations make comparisons between these findings a challenge. Nonetheless, the multilevel growth curve approach is more robust and recommended for longitudinally structured data [42].

Taken together, there is equivocal evidence on the potential relationships between sedentary behaviour and bodily pain. However, our finding from a large cohort of community-based middle-aged and older adults does corroborate some of the existing evidence on detrimental associations; specifically, our finding that time spent sitting and watching television predicts the severity of bodily pain at a given time point of pain trajectory supports the growing public health concerns of excessive sedentary behaviour.

The mechanisms that may underpin the reporting of pain severity are likely to be complex, potentially involving the interplay of biological and multiple psychosocial factors [45, 46]. There is, however, evidence that suggests some behavioural attributes can modulate pain [47–49]. The potential pain modulation role of sedentary behaviour has been understudied compared to physical activity [47]. For instance, there is evidence indicating that higher levels of physical activity are associated with pain inhibition and reduced pain facilitation [47–49]. Nevertheless, evidence supporting a negative relationship between sedentary behaviour and pain modulation has also been reported [48].

The link between sedentary behaviour and adiposity may be a probable pathway that could explain the association of sedentary behaviour with bodily pain [50]. Adipose tissue is metabolically active, releasing pro-inflammatory cytokines and adipokines that may potentiate inflammatory changes in tissues leading to noxious pain stimuli [51]. Also, sedentary behaviour may directly or indirectly, through its association with obesity, lead to a reduction in physical activity levels [18] and modulate the biomechanical loading pathway of some bodily pain, such as somatic joint pain related to older age [52, 53]. In the context of this study, it is important to note that our analysis accounted for the potential confounding bias of adiposity (waist circumference) and physical activity. The observed associations of TV time (sedentary behaviour) with bodily pain, therefore, provide informative evidence on the potential role of sedentary behaviour in the pathogenesis of bodily pain. This may be mediated through some of the known sedentary behaviour associations, for example, with systemic inflammation and vascular endothelial dysfunction, especially in those with metabolic disorders such as T2D [54, 55]; and, plausibly through unknown mechanisms related to a negative modulation influence of sedentary behaviour on pain perception [48].

We observed that those with T2D, especially known T2D (and more likely longer diabetes duration) experienced relatively higher pain severity (Fig. 2) and had slightly higher TV time than those without T2D (Fig. 3). Generally, however, there were only small variations in the bodily pain scores and/or TV time across the three data time-point analysed. These limited variations may have contributed to the observed statistically non-significant or marginally non-significant TV time/T2D status interaction terms. Nevertheless, our findings have shown that compared to those with NGM, the association of increasing TV time with the severity of bodily pain at any given time point is more pronounced in those with T2D than with prediabetes. These observations support the evidence that people with T2D, especially those with long-standing cases, are predisposed to heightened pain due to systemic inflammatory response and vascular complications associated with peripheral neuropathy in T2D [26, 27]. Moreover, compared to those without T2D, people with T2D tend to spend more time in sedentary behaviour [29]. In line with our findings, the higher TV time in those with T2D could partly account for the severe bodily pain observed in this group, as demonstrated in this study. This is consistent with the existing evidence of adverse associations of high TV time with chronic health outcomes, including chronic pain [12–16].

The findings may have some implications in light of the public health and clinical challenges of chronic pain [5]. Aside from the challenges of pharmacologic management of chronic pain, many adults who experience chronic pain are physically inactive [7, 8]. There are some clinical instances where some people who present with bodily pain may be counselled to take regular rest breaks; however, evidence suggests increased activities level improve bodily pain in most people. Though clinical guidelines for chronic pain management have not specifically referred to limiting sedentary behaviour, the importance of physical therapy (which can include exercise prescriptions) has been widely acknowledged, for example, in the American Society of Anaesthesiologists Task Force on Chronic Pain Management guideline [56]. Thus, advocating for strategies with realistic goals that encourage and support people, especially older adults to move more and break up prolonged sitting (sedentary) behaviours can be of benefit to those with chronic pain, as well as other chronic conditions.

There is sufficient evidence on the pain modulation effect of increased physical activity and reduced sedentary behaviour in adults [47–49]. Also, some evidence indicates that reduced sedentary behaviour is associated with reduced musculoskeletal pain conditions [57, 58]. As demonstrated by our findings, leisure-time sedentary behaviour (TV time) can be detrimentally associated with increasing pain severity with advancing age. These findings could help inform future intervention trials in clinical populations to examine the effect of reducing sedentary behaviour on bodily pain trajectory. Also, further study could explore the effects of the balance or interaction of physical activity and sedentary behaviour on the prediction of bodily pain severity. Taken together, findings from these studies would provide insights relevant to the prescription of sedentary behaviour reduction as a non-pharmacologic intervention and adjuvant therapy in chronic pain management, as well as support for public health initiatives to address sedentary behaviour in addition to physical inactivity in ageing adults. Such future studies may consider using device-measured sedentary behaviour and disease-specific pain instrument to minimise measurement bias.

A key strength of this study is the prospective design, using data collected at three-time points over 12 years, allowing some inferences to be made about causality. Though this study is a posthoc analysis, the bodily pain (outcome) and the TV time (exposure) were measured at all three time points. Another strength is a cohort consisting of a large sample of Australian adults; thus, the findings could be reasonably generalised across middle-aged and older adults. Furthermore, the multilevel growth curve statistical approach is an additional strength of this study. The multilevel growth curve method provides numerous advantages, including the ability to handle missing data as missing at random (MAR), the estimation of the mean baseline bodily pain severity and the rate at which the severity increases with age, the between- and within-individual variations as well as the covariance of the intercept and slope variance, and the ability to make predictions relative to exposure effect (in this case, TV time) [42, 43]. This approach, treating all missing data as MAR should have minimised the impact of loss-to-follow-up on the findings. We replicated our analysis in a sensitivity analysis on only those baseline participants who provided data at both follow-ups and observed similar results with only minor changes in effect sizes, but the trends remained the same (Supplementary file, Table S2 and Figure S2). A further strength is the wide range of data on time-invariant and time-variant covariates which were adjusted for as potential confounders in the analysis.

There are several limitations, and the findings should be interpreted in the context of the following: firstly, this is a secondary analysis in that AusDiab was not primarily designed to specifically address the aims of this study. The bodily pain scores were taken from the SF36 questionnaire, a generic instrument for the quality-of-life assessment of populations and are quite different from other instruments used to measure pain in disease-specific studies. Nevertheless, the SF36 bodily pain scale being self-report with an inherent recall bias of underestimating or overestimating pain has been shown to have acceptable psychometric properties; able to detect changes in pain over time; and has widely been used in population-based research to make comparisons across diverse populations [34]. In clinical populations, however, other disease-specific pain instruments may facilitate enhanced pain severity discrimination compared with the SF36 bodily pain scale [34]. Importantly, it must be acknowledged that bodily pain is heterogeneous, and there might be some pain-related conditions that benefit from sedentary behaviour while others are aggravated by excessive sedentary behaviours. Secondly, the exposure variable (TV time) was self-reported and represented a particular subset of leisure-time sedentary behaviour. Time spent on the internet and social media are examples of other components of overall leisure-time sedentary behaviour, that were not captured. It is important to note here that not accounting for the other leisure-time sedentary behaviour have may potentially led to underestimation or overestimation of the magnitude of TV time associations with the bodily pain severity.

Thirdly, data on some potential time-variant confounders such as a history of cancer and bone fracture were available at only one-time point and assumptions were made to either treat those variables as time-invariant variables if it was measured at only baseline (history of cancer) or exclude those participants (bone fracture) in the analysis to account for potential reverse causation bias. Finally, there could well be other unmeasured confounders, therefore, not accounted for in the analysis. For instance, there are some chronic conditions such as pain disorders of the musculoskeletal system which could influence both sedentary behaviour and pain outcome, but data were not available and hence not accounted for in our analysis. Also, the duration of T2D may have had an impact on the findings but this was not assessed in the study. However, cardiovascular conditions and chronic kidney diseases which are often associated with complications of T2D were accounted for. Future studies could consider examining sedentary behaviour/pain associations exclusively in those with T2D and the potential interactions of the relationships with T2D duration and mobility limitations.

## Conclusions

In this cohort of middle-aged to older Australian adults, we showed that bodily pain increases in severity with ageing; and increasing TV time at any given time point was found to be significantly associated with increased severity of bodily pain. Those with T2D tended to report higher pain levels than those without T2D, and the association of TV time with bodily pain severity at any particular time point was more pronounced in those with T2D than those without T2D. Specifically, compared to those with NGM, the effect of T2D on the severity of bodily pain with increasing TV time was significantly pronounced when the TV time threshold increased above 2.5 hours per day, but that of prediabetes was statistically non-significant. Considering the available evidence on the pain modulation effect of physical activity, our findings align with the WHO’s physical activity and sedentary behaviour recommendation guidelines [59] of increasing levels of moderate-to-vigorous intensity physical activity and also reducing time spent in sedentary behaviours. Controlled intervention trials in disease-specific clinical populations to examine the effect of reducing prolonged sedentary behaviour on bodily pain in the long term will provide stronger support for clinical and public health initiatives to reduce sedentary time, as well as some evidence on non-pharmacologic benefits of sedentary behaviour reduction and a potential adjuvant pain modulation therapy for chronic pain management guidelines.

## Supplementary Information

**Additional file 1.**

## Data Availability

The data that support the findings of this study are available from the AusDiab Steering Committee, but restrictions apply to the availability of these data, which were used under license for the current study, and so are not publicly available. Data are, however, available upon reasonable request by written applications to the AusDiab Steering Committee (Dianna.Magliano@baker.edu.au).

## Abbreviations

CVD: Cardiovascular disease
METs: Metabolic equivalents
T2D: Type 2 diabetes
TV: Television-viewing
SF-36: 36-Item Short-Form Health Survey questionnaire
AusDiab: Australian Diabetes, Obesity, and Lifestyle Study
CCD: Census Collector District
OGTT: Oral glucose tolerance test
IFG: Impaired fasting glucose
IGT: Impaired glucose tolerance
NGM: Normal glucose metabolism
HRQoL: Health-related quality of life
DVD: Digital Video Disc
ICC: Intraclass (correlation) coefficient
WHO: World Health Organisation
FBG: Fasting blood glucose
ADA: American Diabetes Association
AAS: Active Australia Survey
MVPA: Moderate-to-vigorous physical activity
CKD: Chronic kidney disease
VIF: Variance Inflation Factor
SE: Standard error
MAR: Missing at random
