# Supplementary material for: Television-viewing time and bodily pain in Australian adults with and without type 2 diabetes: 12-year prospective relationships
Source: BMC Public Health. 2022 Nov 29;22:2218. doi: 10.1186/s12889-022-14566-y (PMC9706940; doi:10.1186/s12889-022-14566-y)
Supplement: Supplementary file 1 — Additional file 1. [file 12889_2022_14566_MOESM1_ESM.docx]

**Supplementary File**

**Television-Viewing Time and Bodily Pain in Australian Adults with and without Type 2 Diabetes: 12-Year Prospective Relationships**

**Authors:**

Francis Q. S. Dzakpasu, Neville Owen, Alison Carver, Parneet Sethi, Christian J. Brakenridge, Agus Salim, Donna M. Urquhart, Flavia Cicuttini, and David W. Dunstan

**Statistical method: Multilevel linear growth curve model**

Multilevel growth curve modeling is robust in handling missing data by treating them as missing at random, minimizing bias in estimates attributable to attrition in longitudinally structured data[[1](#_ENREF_1), [2](#_ENREF_2)]. Also, the incorporated multilevel approach in the growth curve model accounted for the complex survey design of the AusDiab. A three-level linear growth curve model was fitted with the repeated measures at level-1 nested in individuals at level-2 who are also nested in CCD clusters at level-3.

First, unconditional growth trajectory was modeled (random intercept and random slope models) with bodily pain score regressed as a function of age (centered at age 50 years, about the mean age at baseline). A likelihood ratio test comparing the two models indicated that the more flexible random slope model with unstructured covariance was preferred over the random intercept model (chi-square test (χ^2^) = 22.53; p < 0.001).

The equation for the unconditional multilevel linear growth curve model for individual *i* in cluster *j* at measurement occasion (age) *t* is presented as:

*Level 1: Repeated Measurement within an individual*

$Y_{\left( Bodily pain \right)ijt}= \pi_{0ij}+ \pi_{1ij}{Age}_{it}+ \varepsilon_{ijt}$ *[* $\varepsilon_{ijt}\sim N(0,\sigma_{\varepsilon}^{2})$ *]*

*Level 2: Individual nested within cluster (CCD)*

$\pi_{0ij}= \beta_{0j}+ \mu_{0ij}$ *[* $\mu_{0ij}\sim N(0,\sigma_{\mu_{0}}^{2})$ *]*

$\pi_{1ij}= \beta_{1j}+ \mu_{1ij}$ *[* $\mu_{1ij}\sim N(0,\sigma_{\mu_{1}}^{2})$ *]*

*Level 3: Cluster*

$\beta_{0j}= \gamma_{0}+ \nu_{0j}$ *[* $\nu_{0j}\sim N(0,\sigma_{\nu_{0}}^{2})$ *]*

*Or in composite*

$$Y_{\left( Bodily pain \right)ijt}=\gamma_{0}+\beta_{1j}{Age}_{it}+[ \nu_{0j} +\mu_{0ij}+\mu_{1ij}{Age}_{it}] + \varepsilon_{ijt}$$

*Where,*

$\varepsilon_{ijt}\sim N(0,\sigma_{\varepsilon}^{2})$

$\left( \begin{matrix} \mu_{0ij} \\ \mu_{1ij} \end{matrix} \right) \sim N\left( 0, \Omega_{\mu} \right), where \Omega_{\mu}= \left( \begin{matrix} \sigma_{\mu_{0}}^{2} & \\ \sigma_{\mu_{0}\mu_{1}} & \sigma_{\mu_{1}}^{2} \end{matrix} \right)$ *and*

$\nu_{0j} \sim N\left( 0, \sigma_{\nu_{0}}^{2} \right)$

*Where* $\pi_{0ij}$ *+* $\pi_{1ij}{Age}_{it}$ *is mean bodily pain for individual i in cluster j at age t and* $\varepsilon_{ijt}$ *is the difference between the observed bodily pain for this individual i and the mean.* $\beta_{0j}$*is the mean bodily pain at age=0 across all individuals in cluster j, while* $\mu_{0ij}$ *measures how much the mean bodily pain at age=0 for individual i differs from their cluster-level average. Similarly,* $\beta_{1j}$*is the mean change in bodily pain per unit age for all individuals in cluster j, while* $\mu_{1ij}$ *measures how much individual i differs from their cluster-level average in terms of this parameter. Further,* $\gamma_{0}$ *is the mean bodily pain at age=0 averaged across all clusters and* $\nu_{0j}$ *measures how cluster j differs from this average. Finally,* *The variance in the bodily pain due to the clustering of the individual is var*$\left( \nu_{0j} \right)=\sigma_{\nu_{0}}^{2}$*, whereas the between-individual variance in the bodily pain is var*$\left( \mu_{0ij} \right)= \sigma_{\mu_{0}}^{2}$*, between individual variance in the slope is var*$\left( \mu_{1ij} \right)= \sigma_{\mu_{1}}^{2}$*, and the individuals’ intercept-slope covariance is* $\sigma_{\mu_{0}\mu_{1}}$*.*

Note: In the conditional growth curve model, to understand whether the effect of TV time on bodily pain trajectory changed with age, the interaction between TV time and age was added to the fitted model, but the interaction term was non-significant, and a likelihood ratio test comparing this with the previous non-interaction model was non-significant (χ^2^ = 0.14; p = 0.707). Thus, the non-interaction model was preferred over the interaction model. A linear-additive model was therefore fitted, excluding the interaction term.

**References**

1. Gibbons RD, Hedeker D, DuToit S: **Advances in analysis of longitudinal data**. *Annu Rev Clin Psychol* 2010, **6**:79-107.

2. Dingemanse NJ, Dochtermann NA: **Quantifying individual variation in behaviour: Mixed-effect modelling approaches**. *J Anim Ecol* 2013, **82**(1):39-54.

Sensitivity analysis

Two sensitivity analyses were performed.

1. A sensitivity analysis excluding data for those participants who reported a history of cancer to check the robustness of our analysis. Data on participants’ history of cancer was only available at a one-time point (baseline) with the assumption made that it was a time-invariant covariate in the analysis. Secondly, many of those with a history of cancer may be more likely to self-report experiencing more pain. A total of 3,827 participants with complete data at baseline were included in this sensitivity analysis. Results are in Table S1 and Figure S1.
2. A sensitivity analysis on those participants who provided data at baseline and both of the follow-ups was also performed. There were 2,727 such participants in the included study sample. The analytic modelling adjusted for all the covariates as described in the main analysis, including the history of cancer variable. Results are in Table S2 and Figure S2.

| **Table S1: Sensitivity analysis for unconditional and conditional linear growth curve models for bodily pain** | | | | | |
| --- | --- | --- | --- | --- | --- |
|  | **Unconditional model** | **Conditional models** | | | |
|  | **Model 1**  **(Function of age)** | **Model 2**  **Model 1 + TV time** | **Model 3**  **Fully adjusted linear-additive model** | **Model 4**  **Model 2 + TV time # T2D status** | **Model 5**  **Fully adjusted with TV time#T2D status** |
|  | ***Coefficient (S.E)*** | ***Coefficient (S.E)*** | ***Coefficient (S.E)*** | ***Coefficient (S.E)*** | ***Coefficient (S.E)*** |
| **Fixed effect** |  |  |  |  |  |
| Intercept | 75.58 (0.45)*** | 77.49 (0.53)*** | 75.96 (2.26)*** | 77.78 (0.55)*** | 75.74 (2.26)*** |
| Slopes |  |  |  |  |  |
| Age (Centred at 50 years) | - 0.27 (0.03)*** | - 0.24 (0.03)*** | - 0.30 (0.03)*** | - 0.21 (0.03)*** | - 0.30 (0.03)*** |
| TV time |  | - 1.11 (0.18)*** | - 0.66 (0.17)*** | - 0.98 (0.20)*** | - 0.50 (0.20)** |
|  |  |  |  |  |  |
| T2D status |  |  |  |  |  |
| NGM (Reference) |  |  |  | 0 | 0 |
| Prediabetes |  |  |  | - 0.67 (0.99) | 1.26 (0.99) |
| T2D |  |  |  | - 4.54 (1.54)** | 0.19 (1.52) |
|  |  |  |  |  |  |
| TV time#T2D status |  |  |  |  |  |
| NGM (Reference) |  |  |  | 0 | 0 |
| Pre-diabetes |  |  |  | - 0.25 (0.42) | - 0.40 (0.41) |
| T2D |  |  |  | - 0.52 (0.57) | - 0.91 (0.56) |
|  |  |  |  |  |  |
| **Random effect** |  |  |  |  |  |
| Intercept variance |  |  |  |  |  |
| Cluster^#^ | 4.71 (1.93)** | 4.01 (1.77)** | 1.07 (0.92) | 3.37 (1.61)** | 1.06 (0.92) |
| Participants | 191.92 (7.65)*** | 190.17 (7.63)*** | 143.87 (6.73)*** | 187.78 (7.64)*** | 145.05 (6.05)*** |
| Slope variance | 0.012 (0.006)** | 0.013 (0.006)** | 0.006 (0.004) | 0.013 (0.006)** | 0.006 (0.004) |
| Intercept-Slope covariance | 1.51 (0.35)** | 1.55 (0.35)** | 0.93 (0.31) | 1.54 (0.35)* | 0.93 (0.31) |
|  |  |  |  |  |  |
| Within-individual variance | 264.90 (4.72)*** | 264.50 (4.75)*** | 261.63 (4.87)*** | 264.34 (4.74)*** | 261.44 (4.87)*** |
|  |  |  |  |  |  |
| **Goodness-of-fit** |  |  |  |  |  |
| AIC | 89576.09 | 88768.87 | 84666.83 | 88685.66 | 84667.67 |
| BIC | 89590.54 | 88790.51 | 84824.70 | 88736.16 | 84839.90 |
| Log-likelihood | - 44786.05 | - 443841.44 | - 42311.41 | - 44335.83 | - 42309.84 |
| No of parameters | 7 | 8 | 22 | 12 | 24 |
| Statistically significant: *** p < 0.001; ** p < 0.01; ** p < 0.05 \| TV time#T2D status: Interaction between TV time and T2D status \| TV = Television-viewing \| S.E = Standard error \| NGM = Normal glucose metabolism \| T2D = Type 2 diabetes (included newly diagnosed and known T2D).  The analysis excluded 6.6% of participants that reported a history of cancer at baseline (this data was only available at baseline)  The fully adjusted linear additive model included model 2 + sex, education level, household income, smoking status, leisure-time physical activity, waist circumference, energy intake, T2D status, SF36 mental component score, presence of chronic kidney disease, and history of cardiovascular disease (CVD).  The fully adjusted model with TV time#T2D status included model 4 + sex, education level, household income, smoking status, leisure-time physical activity, waist circumference, energy intake, SF36 mental component score, presence of chronic kidney disease, and history of cardiovascular disease (CVD).  ^#^ This represents the intercept variance that is attributable to the level 3 clustering of individuals (individuals nested in clusters); thus, describes the variance component of cluster-to-cluster variability. | | | | | |

| **Table S2: Sensitivity analysis for unconditional and conditional linear growth curve models for bodily pain** | | | | | |
| --- | --- | --- | --- | --- | --- |
|  | **Unconditional model** | **Conditional models** | | | |
|  | **Model 1**  **(Function of age)** | **Model 2**  **Model 1 + TV time** | **Model 3**  **Fully adjusted linear-additive model** | **Model 4**  **Model 2 + TV time # T2D status** | **Model 5**  **Fully adjusted with TV time#T2D status** |
|  | ***Coefficient (S.E)*** | ***Coefficient (S.E)*** | ***Coefficient (S.E)*** | ***Coefficient (S.E)*** | ***Coefficient (S.E)*** |
| **Fixed effect** |  |  |  |  |  |
| Intercept | 75.86 (0.46)*** | 77.71 (0.55)*** | 78.38 (2.57)*** | 77.80 (0.58)*** | 78.03 (2.58)*** |
| Slopes |  |  |  |  |  |
| Age (Centred at 50 years) | - 0.26 (0.03)*** | - 0.23 (0.03)*** | - 0.29 (0.03)*** | - 0.21 (0.03)*** | - 0.29 (0.03)*** |
| TV time |  | - 1.10 (0.19)*** | - 0.71 (0.19)*** | - 0.95 (0.22)*** | - 0.49 (0.22)* |
|  |  |  |  |  |  |
| T2D status |  |  |  |  |  |
| NGM (Reference) |  |  |  | 0 | 0 |
| Prediabetes |  |  |  | - 0.55 (1.11) | 1.70 (1.11) |
| T2D |  |  |  | - 1.96 (1.71) | 1.96 (1.67) |
|  |  |  |  |  |  |
| TV time#T2D status |  |  |  |  |  |
| NGM (Reference) |  |  |  | 0 | 0 |
| Pre-diabetes |  |  |  | - 0.16 (0.46) | - 0.51 (0.46) |
| T2D |  |  |  | - 1.13 (0.64) ^$^ | - 1.44 (0.62)* |
|  |  |  |  |  |  |
| **Random effect** |  |  |  |  |  |
| Intercept variance |  |  |  |  |  |
| Cluster^#^ | 3.59 (1.91) | 3.38 (1. 85) | 1.12 (1.10) | 3.03 (1.76) | 1.14 (1.11) |
| Participants | 184.53 (8.05)*** | 182.62 (8.46)*** | 142.14 (7.63)*** | 181.83 (8.00)*** | 142.06 (7.62)*** |
| Slope variance | 0.011 (0.002)*** | 0.012 (0.006)*** | 0.025 (0.057) | 0.011 (0.002)*** | 0.024 (0.057) |
| Intercept-Slope covariance | 1.43 (0.11)*** | 1.48 (0.37)*** | 0.57 (0.44) | 1.44 (0.11)*** | 0.58 (0.43) |
|  |  |  |  |  |  |
| Within-individual variance | 259.79 (5.02)*** | 259.65 (5.03)*** | 255.13 (5.56)*** | 259.36 (5.05)*** | 254.94 (5.56)*** |
|  |  |  |  |  |  |
| **Goodness-of-fit** |  |  |  |  |  |
| AIC | 71611.07 | 70942.77 | 67585.98 | 70931.35 | 67584.16 |
| BIC | 71660.10 | 70998.72 | 67745.92 | 71015.29 | 67758.01 |
| Log-likelihood | - 35798.53 | - 35463.38 | - 33769.99 | - 35453.68 | - 33767.08 |
| No of parameters | 7 | 8 | 23 | 12 | 25 |
| Statistically significant: *** p < 0.001; ** p < 0.01; * p < 0.05; ^$^ p = 0.076 \| TV time#T2D status: Interaction between TV time and T2D status \| TV = Television-viewing \| S.E = Standard error \| NGM = Normal glucose metabolism \| T2D = type 2 diabetes (included newly diagnosed and known T2D)  The fully adjusted linear additive model 3 included model 2 + sex, education level, household income, smoking status, leisure-time physical activity, waist circumference, energy intake, T2D status, SF36 mental component score, presence of chronic kidney disease, history of cardiovascular disease (CVD), and cancer.  The fully adjusted model 5 with TV time#T2D status included model 4 + sex, education level, household income, smoking status, leisure-time physical activity, waist circumference, energy intake, SF36 mental component score, presence of chronic kidney disease, history of cardiovascular disease (CVD), and cancer.  ^#^ This represents the intercept variance that is attributable to the level 3 clustering of individuals (individuals nested in clusters); thus, describes the variance component of cluster-to-cluster variability. | | | | | |

**
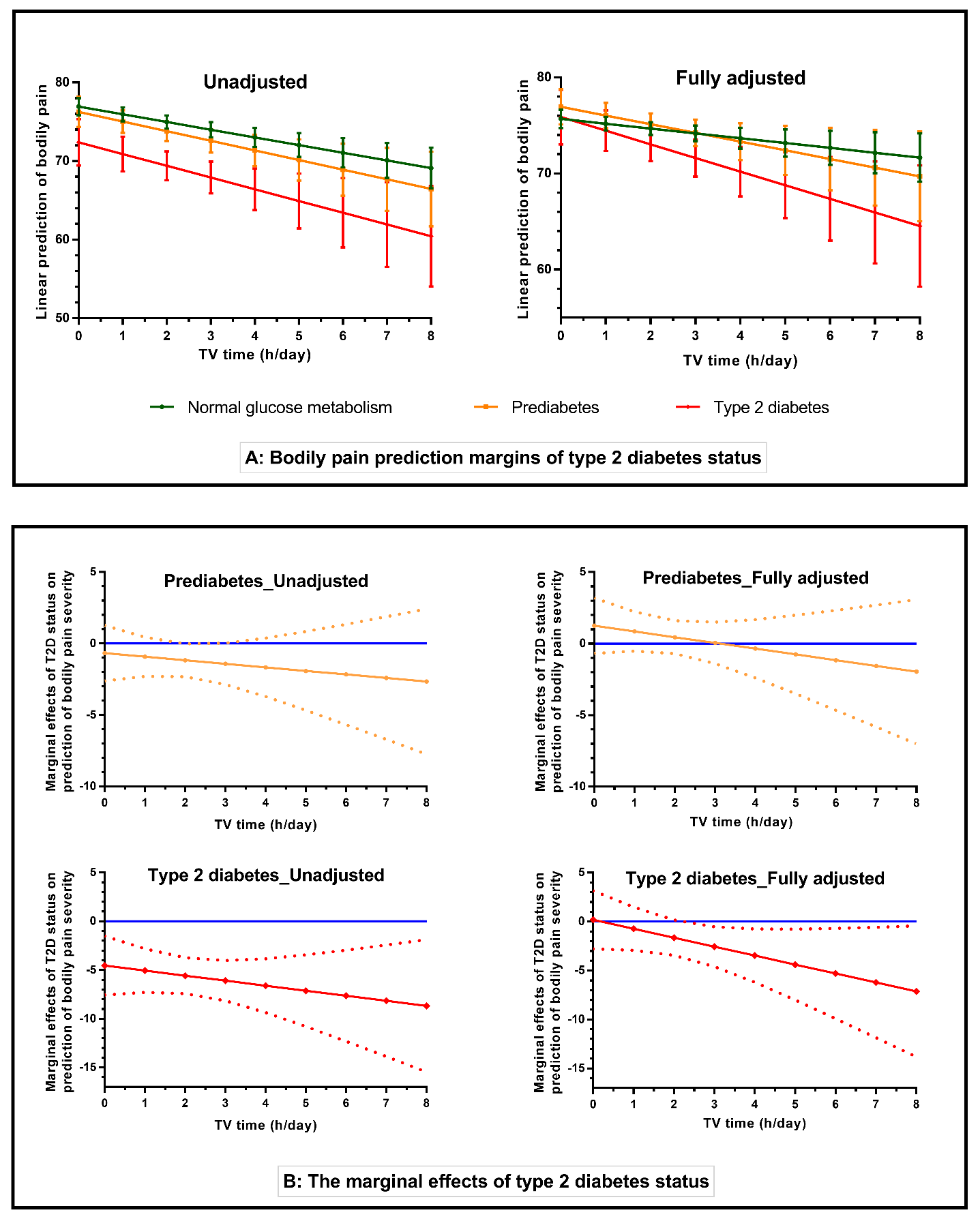
**

**Figure S1:** Shows the sensitivity analysis after excluding those with a history of cancer to check the robustness of the relationships of TV time with bodily pain severity and potential moderation of type 2 diabetes status. **(A)** The bodily pain prediction margins of type 2 diabetes status with 95% confidence intervals for the unadjusted and fully adjusted models. **(B)** The marginal effects of prediabetes and type 2 diabetes (in reference to normal glucose metabolism) on bodily pain severity at different thresholds of TV time for the unadjusted and fully adjusted models. The solid lines indicate the marginal effects of changes in bodily pain severity with changing TV time. The dotted lines are the confidence intervals around the lines, which determine the threshold of TV time that has a statistically significant effect on bodily pain severity in those with prediabetes (ORANGE) and type 2 diabetes (RED). They are statistically significant whenever the lower and upper limits of the confidence intervals are both below or above the zero (0 - BLUE) lines. Note: Normal glucose metabolism was set as the reference point in the regression model.


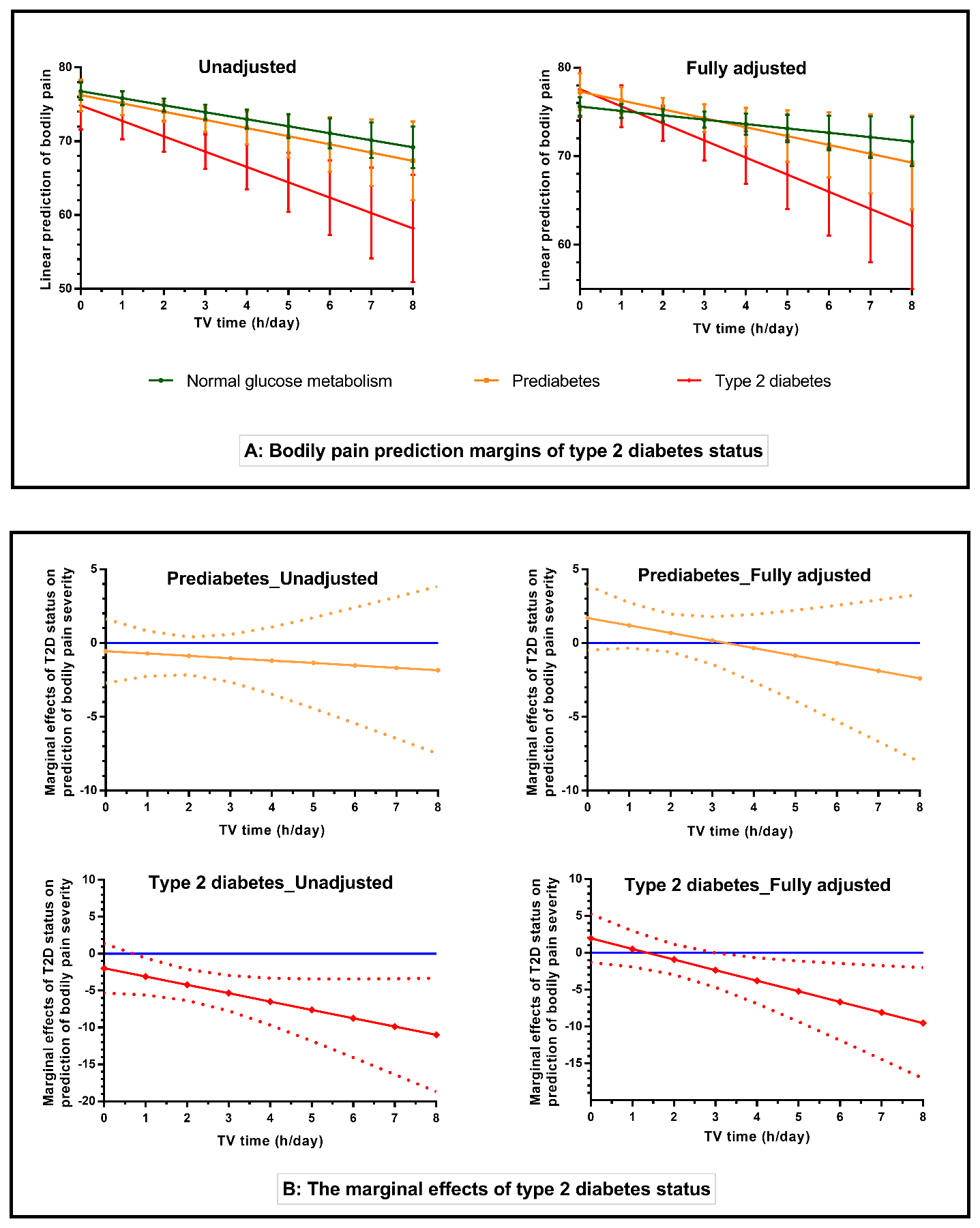


**Figure S2:** Shows the sensitivity analysis of those participants who provided data at baseline and each of the respective follow-ups to check the robustness of the relationships of TV time with bodily pain severity and potential moderation of type 2 diabetes status. **(A)** The bodily pain prediction margins of type 2 diabetes status with 95% confidence intervals for the unadjusted and fully adjusted models. **(B)** The marginal effects of prediabetes and type 2 diabetes (in reference to normal glucose metabolism) on bodily pain severity at different thresholds of TV time for the unadjusted and fully adjusted models. The solid lines indicate the marginal effects of changes in bodily pain severity with changing TV time. The dotted lines are the confidence intervals around the lines, which determine the threshold of TV time that has a statistically significant effect on bodily pain severity in those with prediabetes (ORANGE) and type 2 diabetes (RED). They are statistically significant whenever the lower and upper limits of the confidence intervals are both below or above the zero (0 - BLUE) lines. Note: Normal glucose metabolism was set as the reference point in the regression model.
